# Insights into Molecular Interactions of human Wnt5b and Frizzled proteins for their role in teratogenicity

**DOI:** 10.6026/97320630015246

**Published:** 2019-04-15

**Authors:** Sween Dahiya, Vandana Saini, Pawan Kumar, Ajit Kumar

**Affiliations:** 1 Toxicology and Computational Biology Group, Centre for Bioinformatics, M. D. University, Rohtak, Haryana 124001 India

**Keywords:** Frizzled, homology modeling, molecular docking, fetal development

## Abstract

Wnt-Fzd signalling plays vital role in different physiological pathways including embryonic development and supposed to be probable target of many teratogens. The present study was done to investigate the role of human Wnt5b interaction with different isoforms of human Fzds and also the molecular interactions of their complexes with selected known teratogens [Carbamazepine (CBZ), Retinoic acid (RA), Valproic acid (VPA), Aminopterin (AMP) and Phenytoin (PHY)] using Niclosamide (NLM) as standard. The models of hWnt5b and hFzd isoforms, whose solved crystal structures were unavailable, were generated using homology modeling and hWnt5b was subjected to protein-protein docking studies against different isoforms of hFzd. The macromolecular docking studies of hWnt5b-hFzds complexes revealed that hWnt5b had highest binding affinity with hFzd8 and lowest with hFzd1, respectively. The Cysteine rich domain (CRD) of hFzds docked against hWnt5b into a palm shaped opening or near the largest binding pocket as in hWnt5b-hFzd6. The possible role of Wnt-Fzd interactions in developmental toxicity due to selected teratogens were also investigated using molecular docking studies which showed that Retinoic Acid possessed the maximum binding affinity with binding energy of for hWnt5b-hFzd8 complex while VPA was observed to have lowest binding affinity towards all the studied hWnt5b-hFzd complexes.

## Background

Wnt/Fzd signaling plays significant role in human development and physiology. They are reportedly observed to regulate embryonic development processes like stem cell differentiation, organogenesis, patterning etc 1. Wnts belong to a large family of secretory proteins and are evolutionarily conserved. They have been found to be majorly involved in development of both invertebrates and vertebrates. Frizzled (Fzd) is a seven transmembrane domain spanning protein receptor having a cysteine rich domain (CRD) outside the membrane. CRD is basically known to bind with the Wnt proteins and activate Wnt/Fzd signaling pathways 2, 3. Wnts are found to interact with multiple receptors and carry out different Wnt signaling pathways. The interaction information for different isoforms of Wnt in mammals is very elusive and limited and still not so much explored 4, 5. Out of different isoforms, Wnt5b has been predicted to cause type II diabetes and also to have role in adipogenesis 6 and insulin secretion 7. In E. coli, Wnt5b has been shown to activate Gα° protein along with Fzd1 and Fzd6 8. Wnt5b has also been reported to be expressed in the majority of esophageal cancer cell lines and up-regulated by β-estradiol in MCF-7 cells derived from breast cancer 9. Secreted Wnts are hydrophobic in nature and associated with membrane. There is a paucity of crystal structures identification of Wnt proteins due to its hydrophobicity 10. Homology modeling has always been a tool of choice for evolutionarily related proteins, whose crystal structures are difficult to be deciphered 11. So, the present study was undertaken to have an in-silico molecular level insights into the interactions and signaling of human Wnt5b (hWnt5b) with different human Frizzled (hFzd) proteins. To achieve this objective, the models of hWnt5b and hFzd isoforms, whose solved crystal structures were unavailable, were generated using homology modeling and hWnt5b was subjected to molecular docking studies against different isoforms of hFzd. 

## Methodology

The crystal structure solutions of hWnt5b and isoforms of hFzd (hFzd1, hFzd2, hFzd3 and hFzd6) were not available in Protein Databank (PDB) and hence, their models were constructed using homology modeling method. The models thus generated were refined and evaluated. The energy minimized structures were used for further docking studies. 

### Template search for homology modeling

Protein sequences of hWnt5b and selected hFzds (hFzd1, hFzd2, hFzd3 and hFzd6) were retrieved from NCBI and Swiss-Prot and subsequently subjected to BLASTp against PDB database. Sequences with maximum sequence identity and query coverage were selected as respective template for further homology modeling. 

### Homology modeling and model validation

The homology modelling of hFzds was done using MODELLER version 9.15 12. Different steps of comparative modelling were performed using different python scripts, viz., format.py for conversion of format from FASTA to PIR; align2D.py for templatetarget alignment; model.py for model building and evaluation.py for model evaluation. The 3-D homology model of hWnt5b was generated on the selected template using online Swiss-Model tool in template mode 13 as the quality of models generated by MODELLER version 9.15 was relatively lower and unacceptable for further studies. 

#### Model validation and Energy minimization

Best models of hFzds were selected on the basis of Dope score and GA341 score. The model validation was done by using different structure assessment tool of SWISS-MODEL 14 to assess different attributes of the model qualities. Ramachandran plot was plotted by using Procheck tool, QMEAN6 15 and QMEAN Z-score were also calculated to analyze the overall quality of the models. Verfy- 3D 16 and Errat 17 score were also computed to validate constructed structures. Modelled structures after validation were then subjected to energy minimization using Chimera version 1.11 18. Hundred steps of steepest descent and conjugate gradient methods of energy minimization were performed one by one for both hWnt5b and hFzds (hFzd1, hFzd2, hFzd3 and hFzd6). Amber ff12SB force field was applied to standard residues and AMI-BCC was employed to the non standard residues of the validated structures to attain global minima. 

Protein-Protein complex formation of hWnt5b-hFzds The energy minimized structures and other selected structures of hFzds including CRD domain of hFzd4, hFzd5, hFzd7 and hFzd8, as obtained from PDB, were subjected to protein-protein docking with hWnt5b to predict the interactions between the both the proteins. Protein-protein dockings of hWnt5b-hFzds were carried out using Hex v8.0 19 by applying Spherical Polar Fourier Transform algorithm. Interacting domain residues of docked proteins were analyzed using Ligplot+ software 20. 

#### Docking simulations of hWnt5b-hFzd complexes with known teratogens

hFzds binding at different pockets and having minimum energy value were undertaken for molecular docking simulations against known teratogens � Carbamazepine (CBZ) 21, Retinoic acid (RA) 22, Valproic acid (VPA) 23, Aminopterin (AMP) 24 and Phenytoin (PHY) 25, using software Autodock v4.2.5.1 26 and considering Niclosamide (a non-teratogen and Wnt-Fzd inhibitor 27, 28) as a positive standard. The molecular structures of selected teratogens and standard were obtained from Pubchem in .mol2 file format. The docked protein-protein complexes of hWnt5b-hFzds were used as receptors for molecular docking simulation with the selected teratogens and standard as ligands. The interacting residues and binding energies were noted for further analyses. Prior to molecular docking, polar hydrogen atoms were added to the receptor complexes of hWnt5b-hFzds followed by adding Gastegier and Kollman charges. AutoGrid 4.2 module of AutoDock v4.2, was used to produce grid maps and by setting the spacing between grid points to default value of 0.375 Å. The grid box size was set at 135 Å, 210 Å and 190 Å (x, y and z-axis) to include every amino acid residue present in rigid macromolecule for blind docking. A total of 50 independent runs per ligand and a step size of 0.2 Å for translations and 5 Å for orientations and torsions and initial population of random individuals with a population sizes of 150 individuals were set as fixed parameters for all the docking analysis using genetic algorithm (GA). For GA, the maximum number of energy evaluation was set to 2500000, maximum number of generations was set to 27000, and maximum number of top individual that automatically survived was set to 1 with the mutation rate of 0.02 and crossover rate of 0.8. The Lamarckian Genetic Algorithm was chosen for generating the best conformer and other docking parameters were set as default. 

### Results

The present study was carried out to decipher hWnt5b structural features and its interactions with hFzds, using in-silico tools and techniques. Also, the role of hWnt5b-hFzd interactions in teratogenicity was also attempted against common teratogens. To predict the atomic features of a protein, like energy involved or binding interactions, the computational techniques such as homology modeling and molecular docking simulation techniques were used in our study. 

### Template selection

The crystal structure of Xenopus Wnt8 in complex with the CRD of Fzd 8 (Chain B) with PDB ID 4F0A 29 was selected as template for homology modeling of hWnt5b as it showed maximum sequence coverage and percent identity of 81% and 39% respectively against query sequence of hWnt5b in BLASTp homology search against PDB database. Similarly, based on BLASTp homology search, crystal structure of CRD of Fzd 8 with PDB ID 5T44 was selected as template for homology modeling of the CRD domain of hFzd1, hFzd2 and hFzd6 with respective percent identity of 91%, 95% and 43%, while crystal structure of CRD of hFzd 5 in complex with PAM with PDB ID 5URY was used as template for hFzd3 with percent identity of 47%. The query coverage of selected hFzds was found to be 100%, 100%, 95% and 92% respectively (Supplementary material 1 available with authors). 

### Homology modeling and Model validation

**Figure 1:**
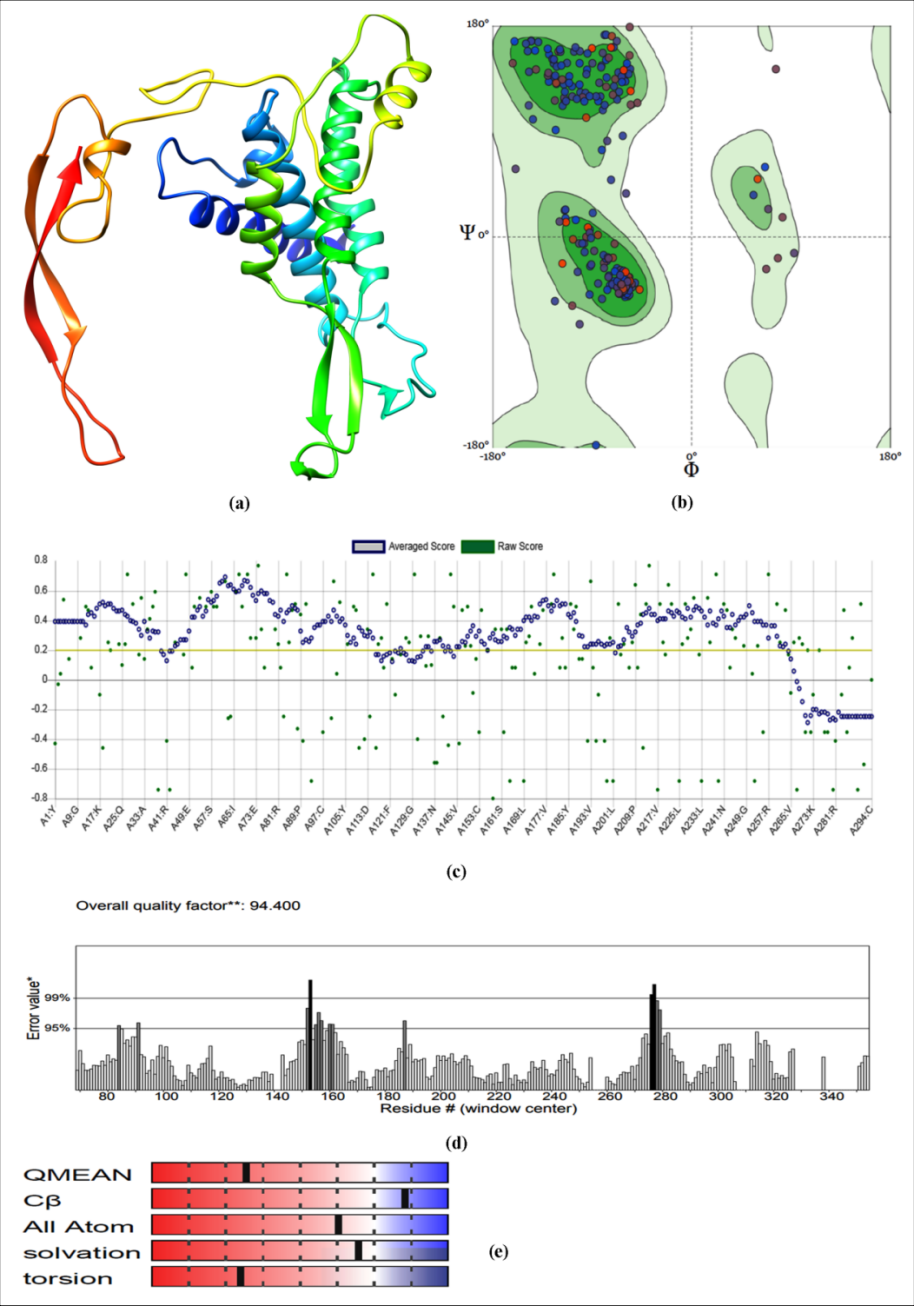
(a) Homology model hWnt5b as generated by SWISS-MODEL using PDB 4F0A_B as template; (b) its Ramachandran Plot, (c) Verify 3D score; (d) ERRAT score as predicted by SAVES and (e) QMEAN Plot as predicted by Swiss Model Assessment tool.

**Figure 2:**
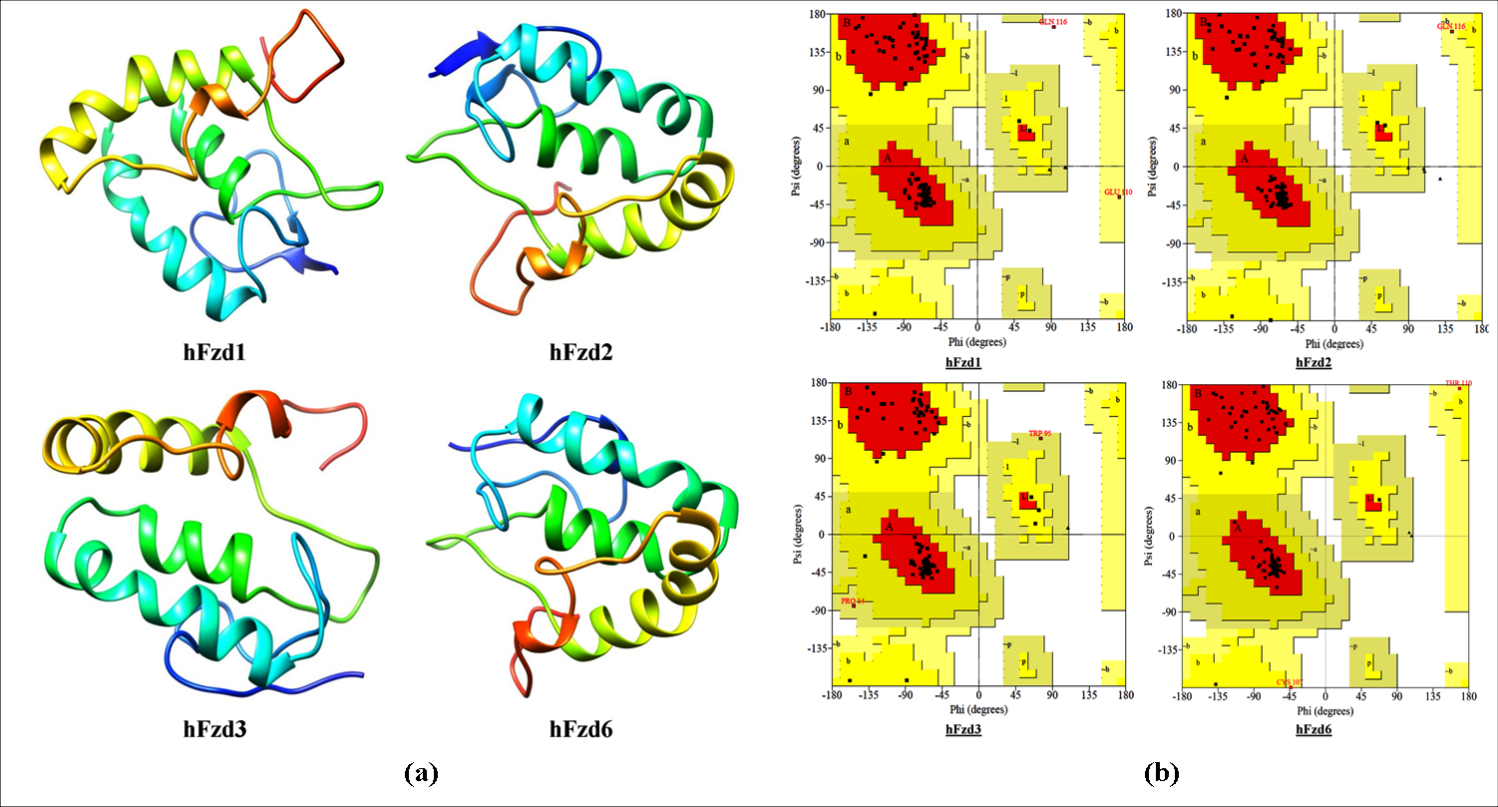
(a) Modeled structure of hFzd1-3 and 6 as generated by MODELLER v9.15 and (b) their corresponding Ramachandran plots as predicted by PROCHECK.

The homology models of selected hFzds (1, 2, 3 and 6) were generated based on selected corresponding templates for hFzds using Modeller v9.15 and Dope scores were calculated for the modeled structure. The modeled structures with minimum Dope scores 30 were selected for further model validation and assessment (Supplementary material 2 - available with the authors). The model of hWnt5b was generated using Xenopus Wnt8 in complex with the cysteine-rich domain of Frizzled 8 (PBD ID 4F0A_B) as template by online SWISS-MODEL server tool. The modeled structures of hWnt5b (Figure 1a) and hFzds (Figure 2a) were validated by analyzing Ramachandran plot predicted by online structure assessment tool of SWISS-MODEL 31. The plots revealed most of the residues of hWnt5b and hFzds in allowed and most generously allowed region (Figure 1b; Figure 2b; Table 1) reflecting optimal overall, residue-by-residue geometry and good stereo chemical quality of the homology modeled protein structures 32. The prediction of crystallographic resolution at which such a quality would be expected of the modeled structure was estimated as Molprobity score 33 that ranged from 1.6 - 3.0 which was quite good value to further use of the modeled structures (Table 1). The Verify 3D score was observed to be greater than 80% for all the fivemodeled structures (Table 1; Figure 1c) thus representing high compatibility of the 3D structures with their primary structures 34. The models of hWnt5b and hFzds were found to be ideal with appropriate overall quality factor and had regions mostly falling in correctly determined region with least non-bonded interactions as predicted by ERRAT (Figure 1d) and QMEAN Plot (Figure 1e). 

**Figure 3:**
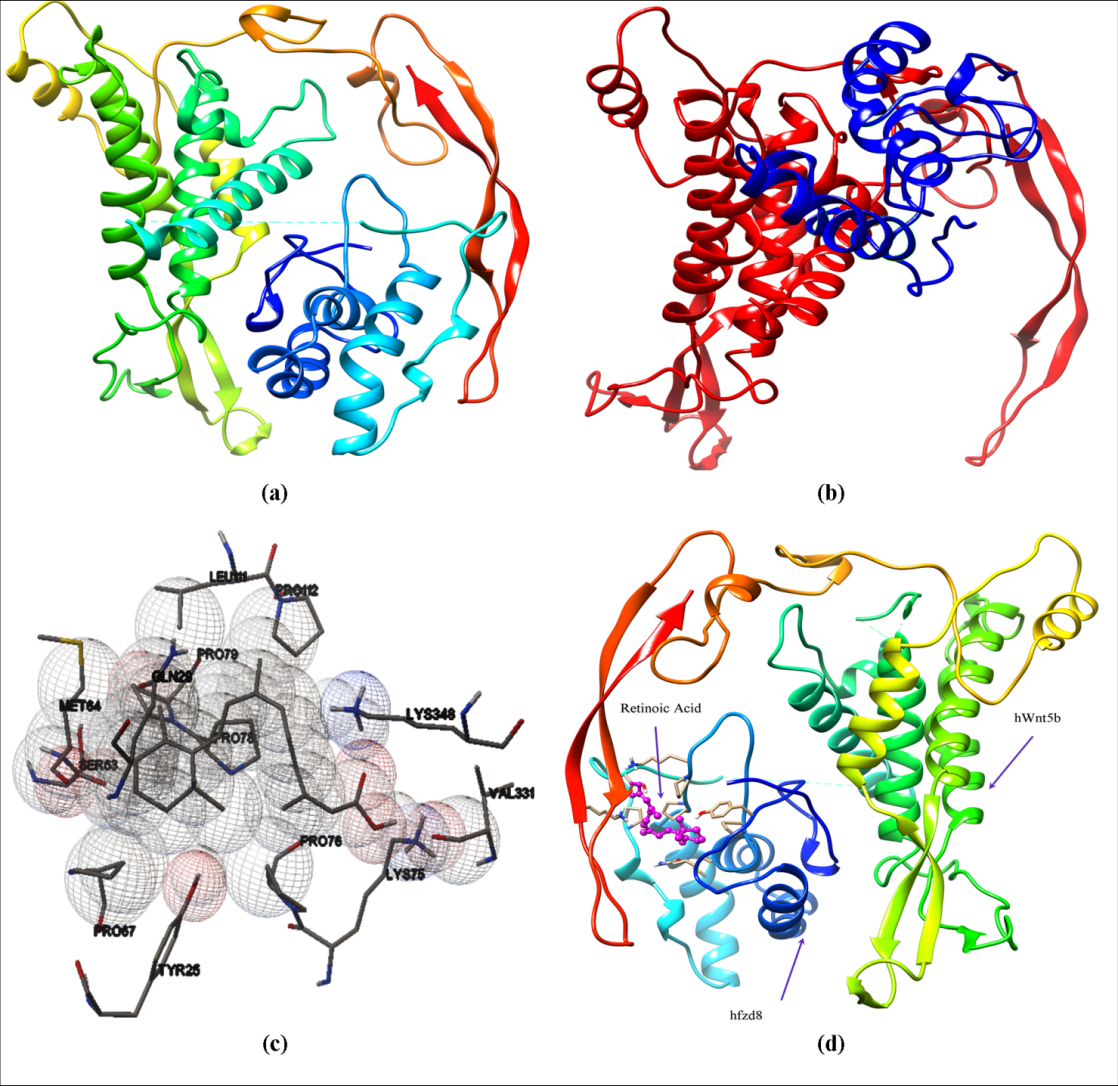
Selected docked complexes of (a) hWnt5b-hFzd6 and (b) hWnt5b-hFzd8 having different binding sites as predicted by HEXv8.0; (c) Interactive residues of hWnt5b-hFzd8 complex with Retinoic acid; (d) Binding site of Retinoic acid when bound with hWnt5b-hFzd8 complex viewed by Chimera version 1.11.

Protein-protein docking of hWnt5b against hFzds The Spherical polar Fourier transform correlation function of Hex v8.0 was used for macromolecular protein-protein docking of hWnt5b against hFzds (hFzd1-hFzd8) to have a quantitative and qualitative insights into hWnt5b-hFzd interactions. The docking analyses illustrated that hWnt5b interacted strongly with hFzd8 possessing highest binding affinity towards hFzd8 with total energy of -625.54 KJ/mol and the energy due to shape of -764.61 kJ/mol. The binding affinities (total energy) of other studied hFzds for hWnt5b were observed to be in the range of -524.48 kJ per mol to -625.54 kJ per mol while the energy due to shape was observed in the range of -509.70 kJ/mol to -764.61 kJ per mol (Table 2). hWnt5bhFzd6 (Figure 3a) and hWnt5b-hFzd8 (Figure 3b) complexes were selected for further docking studies as the former had had highest binding affinity while the later had a different binding pocket as compared to other studied hWnt5b-hFzd complexes (hWnt5bhFzd1; hWnt5b-hFzd2; hWnt5b-hFzd3; hWnt5b-hFzd4; hWnt5bhFzd5 and hWnt5b-hFzd7), which had very subtle differences in binding affinities and binding pockets.

### Molecular Docking Simulations of Wnt5b-Fzds complexes with potent teratogens

The estimation of possible role of Wnt-Fzd interactions in developmental toxicity due to selected teratogens [Phenytoin (PHY); valproic Acid (VPA); carbamazepine (CBZ); retinoic Acid (RA) and aminopterin (AMP)] was carried out by molecular docking studies. The analyses revealed that RA possessed the maximum binding affinity with binding energy of -9.16 Kcal per mol for hWnt5b-hFzd8 complex while VPA was observed to have lowest binding affinity towards all the studied hWnt5b-hFzd complexes (Table 3; Figure 3c-d). 

## Discussion

The current in-silico investigation was performed to gain in-sights into hWnt5b interactions with hFzds and also to interpret the probable function of effective teratogens in impaired neural development by studying their interactions with targeted proteins (hWnt5b-hFzds complexes), the later satisfactorily stated to play major part in neural development processes 35. The unavailable structures of query proteins (hWnt5b, hFzd1, hFzd2, hFzd3 and hFzd6) were built by using homology-modeling approach, while the structures of other selected proteins (hFzd4, hFzd5, hFzd7 and hFzd8) were taken from Protein Data Bank. To improve the quality of the modeled structures, structure validation and energy minimization were also performed. All the models generated were having satisfactory evaluation scores, except for hFzd3 that was found to have non-bonded interactions due to low sequence identity and hence comparatively low Errat score. The overall Zscore of hWnt5b was predicted to be low (-2.827) due to lower resolution and sequence identity of template structure but can be justified as a good working model on the basis of acceptable scores of other quality checks. 

Molecular docking simulations were implicated to predict the molecular orientation and strength of binding of the interacting molecules and their biological role. The relative positions of the interacting molecules influence the vitality and nature of the signal produced by their interaction. Scoring functions used by both protein-protein and protein-ligand docking helps in sampling of the different orientations and conformations of the receptor and ligand molecules and also predicts the exact site of ligand binding and probable site for interacting molecules 36. The macromolecular docking studies of hWnt5b-hFzds complexes revealed that hWnt5b had highest binding affinity with hFzd8 and lowest with hFzd1, respectively, as well as intermediate binding affinity with other studied hFzds. Protein-protein studies clearly showed that CRD of hFzds binds with the hWnt5b into a palm shaped opening or near the largest binding pocket as in hWnt5bhFzd6. The finding is in concurrence with earlier reports where hWnt5b was proved for co-localization with Fzd6 for the formation of Gα° proteins 8. As Wnt and Fzd genes have numerous sites of expression in different systems 37, 38, the macromolecular studies were established as a significant method to uncover the action of binding of proteins at multiple sites and their expression to carry out different processes at the same time. 

The estimation of possible role of Wnt-Fzd interactions in developmental toxicity due to selected teratogens [Phenytoin (PHY); Valproic Acid (VPA); Carbamazepine (CBZ); Retinoic Acid (RA) and Aminopterin (AMP)] was carried out by molecular docking studies. The molecular docking analyses of selected teratogens (PHY, VPA, CBZ, RA and AMP) revealed that RA had maximum binding affinity for hWnt5b-hFzd8 complex, even stronger than the positive standard (NLM) taken for reference, indicating its possible role in affecting Wnt-Fzd pathway during development and causing teratogenicity. The study also revealed that VPA has extensively lower binding affinity towards hWnt7bhFzds complexes, thus hinting on mechanism other than hWnt5bhFzds signaling pathway for its teratogenic behavior. 

## Conclusion

The present study has provided us a good molecular insight into interaction of hWnt5b with hFzd proteins and reveals that hWnt5b binds at similar pocket at the CRD of hFzd1-7 with narrow range of binding energies, while the nature and binding energy of hWnt5b with hFzd8 is different from other isoforms. Also, the study reflects hWnt5b-hFzd8 signaling pathway being interfered as the possible mechanism of teratogenicity due to Retinoic Acid while the case is hinted to be negated for VPA. The study provides a good platform for further inter atomic studies, both at in-silico and in-vitro level. 

## Conflict of Interest

Authors declare no conflict of interest.

## Figures and Tables

**Table 1:** Structure validation of all modeled proteins predicted by structure assessment tool of SWISS-MODEL and SAVES

S. No.	Protein	Whatif Z-score	Molprobity Score	Verify 3D	Errat Score	Ramachandran score (%) Core	Allowed	Disallowed
1	Wnt5b	-2.827	1.69	80.27%	94.4	93.84	1.63	1.63
2	Fzd1	0.553	2.19	99.15%	92.661	93	5	1
3	Fzd2	0.229	2.39	98.31%	81.481	93	6	0
4	Fzd3	0.171	2.88	88.39%	58.654	89.6	9.4	0
5	Fzd6	0.217	2.94	83.04%	75	92.9	5.1	0

**Table 2:** Docking energy and interacting residues profile of hWnt5b docked against selected hFzd proteins

S. No.	Receptor	E-Total (KJ/mol)	E-Shape (KJ/mol)	E-Force (KJ/mol)	No. of H-Bond	Interacting residues
1	Fzd1	-524.48	-569.39	44.91	5	W=GLU74, TYR71, ALA70, GLN66, MET69, LYS81, LYS77, LEU9, ASP98 F= ARG52, TYR40, LYS44, GLN46, PRO8, CYS10, THR11, ASP12
2	Fzd2	-546.9	-591.26	44.37	4	W=LYS81, GLN84, GLU74, PHE326, ARG302, ARG88, LEU303, ASN93, GLU332, GLN330, SER95, LEU297, LYS77, THR96 F= GLN68, PRO21, LYS103, GLN17, TYR1, VAL65, THR64, THR27, ASN28, LEU24, LEU23, GLY25
3	Fzd3	-534.11	-509.7	-24.41	3	W=ARG105, PHE103, GLU292, LEU297, ASN99, VAL106, ALA97 F=GLU37, LEU34, ARG8, THR6, THR31, TYR27, GLN30
4	Fzd4	-588.23	-632.06	43.83	11	W=ASN305, GLN355, SER308, GLN330, VAL353, GLU332, ARG333, LYS348 F=ARG44, CYS45, THR107, GLU108, LYS109, ASP164
5	Fzd5	-546.83	-636.08	89.25	7	W=ARG244, LYS211, GLU276, LYS248, SER251, ASP312, ARG90, LYS246, GLU257, ASP250, ASP243 F=PHE30, ASN31, HIS32, GLU37, GLU41, ARG96, ALA101, GLU104, ARG105, ARG110
6	Fzd6	-592.08	-647.21	55.13	6	W=GLU292, ARG105, SER293, ASN184, LYS187, GLN108, AL97, PHE103, MET69, ARG179, VAL102, GLU182, ASN99 F=VAL7, HIS25, PRO27, GLN29, PHE30, ASN31, LYS75, PRO78, ARG81, ASP109, ARG110, VAL120, LEU111
7	Fzd7	-528.84	-622.02	93.18	4	W=ASN151, GLY125, ASP150, LYS129, GLU126, VAL383, ARG183, GLU187, ALA180, THR130, GLY127, LYS133 F=LEU34, GLN38, PHE39, PRO41, ARG74, PHE91, GLY92, PHE93, GLN94, TRP95, PRO96
8	Fzd8	-625.54	-764.61	139.07	6	W=ASN182, ALA183, GLU190, GLY191, GLU192, ARG186, ALA187, TYR126, GLU129, LYS132, THR133, PHE293, LYS136, HIS140, GLN139, VAL386 F=GLU5, THR7, PRO9, LYS12, GLY13, GLN17, TYR18, CYS64, LEU65, GLU66, ASP67, LYS70, ASP113

**Table 3:** Binding energy and interacting residues of selected hWnt-hFzd complexes with selected teratogens as predicted by Autodock (W: hWnt; F: hFzd)

S.No.	Receptor complex	Ligand	Binding Energy (KJ/mol)	Inhibition constant	H-bond residues	Interacting residues
1	W5bF6	Aminopterin	-5.94	44.43µM	LYS150	TRP232, GLY162, GLY159, LYS150, TRP158, LEU233, ALA147, SER144
2	W5bF6	CBZ	-7.03	7.01 µM	LEU70	PRO76, ILE68, LYS75, TYR73, PRO67, CYS69, ARG105, LEU70, GLU292, HIS74, HIS25
3	W5bF6	Niclosamide	-7.84	1.8 µM	TYR73, LEU121, ASP98	VAL120, LYS81, ASN99, ASP98, GLU119, LEU121, ILE80, GLN84, ALA97, LYS77, TYR73, LYS75
4	W5bF6	Phenytoin	-6.81	10.14 µM	LEU70, ILE68, LYS65	TYR73, LEU70, ARG105, GLU292, CYS69, LYS75
5	W5bF6	Retinoic Acid	-8.16	1.05 µM	VAL113	ASP98, ARG81, ILE72, ASN99, ARG116, MET69, ARG179, VAL113, ALA76, PHE175, PRO112, ASP109, VAL102, SER101
6	W5bF6	VPA	-5.58	81.67 µM	LEU77, ARG105	TYR73, PRO67, LYS75, ILE68, CYS69, ARG105, LEU70, LEU77
7	W5bF8	Aminopterin	-7.23	5.01 µM	PRO27, ASN31, LYS348	ARG333, LYS75, LYS348, TYR25, THR119, ASN31, PRO76, VAL331, LEU6, PRO27, ASN28
8	W5bF8	CBZ	-7.44	3.53	--	TYR98, LEU48, PHE45, PRO47, GLN44, MET95, PHE100
9	W5bF8	Niclosamide	-8.31	808.87nM	THR119, ARG333, LYS348, GLU332	THR119, LYS75, PRO76, GLU332, ARG333, LYS348, VAL331
10	W5bF8	Phenytoin	-7.14	5.87 µM	LYS75, VAL331, LYS74, GLU332	ARG333, LYS75, LYS74, VAL331, GLU332, PRO76
11	W5bF8	Retinoic Acid	-9.16	191.71nM	LYS75, LYS348	MET64, TYR25, PRO67, GLN29, PRO112, LYS348, LEU111, PRO78, PRO79, PRO76, LYS75, VAL331, SER63
12	W5bF8	VPA	-4.88	266.27 µM	LYS75, GLN330, VAL331	LYS75, PRO117, THR119, VAL331, ASP118, GLN330
